# Supplementary material for: PCNA recruits cohesin loader Scc2 to ensure sister chromatid cohesion
Source: Nat Struct Mol Biol. 2023 Aug 17;30(9):1286–94. doi: 10.1038/s41594-023-01064-x (PMC10497406; doi:10.1038/s41594-023-01064-x)
Supplement: Supplementary file 1 — Supplementary Fig. 1 and Tables 1 and 2 [file 41594_2023_1064_MOESM1_ESM.pdf]

nature structural & molecular biology

Article

https://doi.org/10.1038/s41594-023-01064-x

PCNA recruits cohesin loader Scc2 to ensure sister chromatid cohesion

In the format provided by the authors and unedited

Supplementary Figures

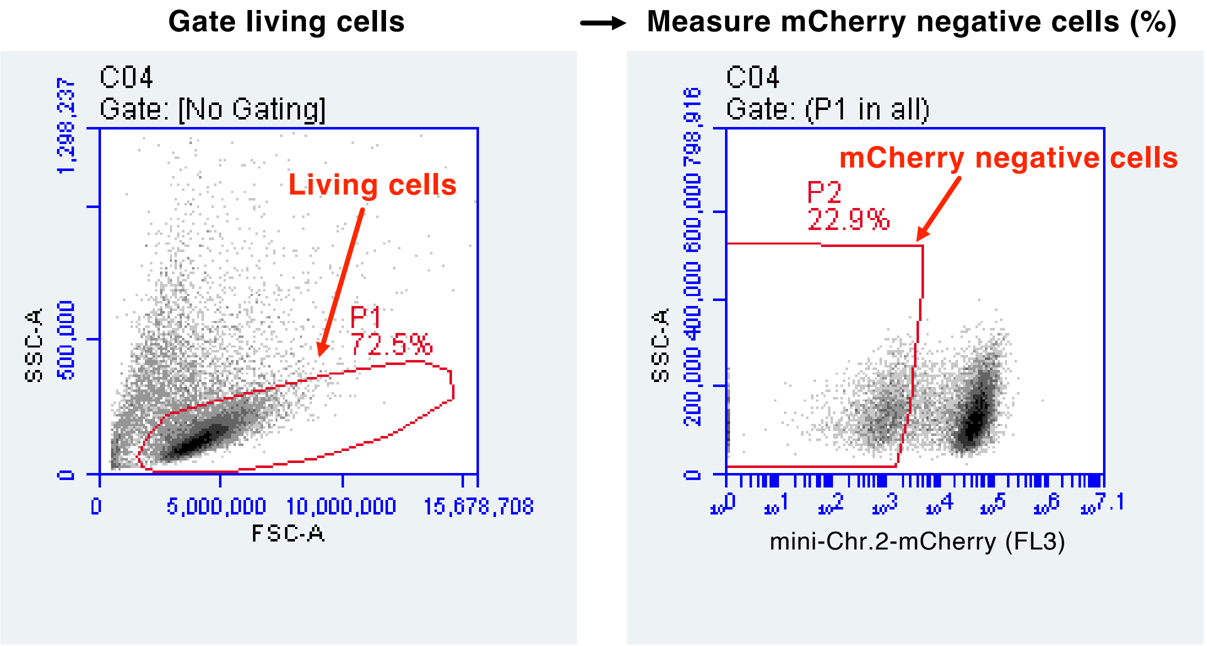

Supplementary Fig. 1: Gating strategy for flow cytometry analysis related to Fig. 6b.

For mini-chromosome loss assay, samples were gated on SSC-A and FSC-A to exclude dead cells and debris. Then living cells (P1) were gated on SSC-A and FL3 to determine the percentage of mCherry negative cells (P2), which correspond to the mini-chromosome loss rate. 10000 of living cells per sample were analysed.

Supplementary Table 1. ***Saccharomyces cerevisiae*** **strains used in this study.**

Strain	Relevant Genotype	Reference
FY1363 (W303 WT)	*MATa ade2-1 can1-100 his3-11,15 leu2-3,112 trp1-1 ura3-1 RAD5+*	Lab collection
FY1364 (W303 WT)	*MATalpha ade2-1 can1-100 his3-11,15 leu2-3,112 trp1-1 ura3-1 RAD5+*	Lab collection
HY10598	*MATa/MATalpha elg1::HIS3MX6/elg1::HIS3MX6 wpl1::TRP1/wpl1::TRP1 PDS5/pds5::NatMX4*	This study
HY10876	*MATa/MATalpha elg1::HIS3MX6/elg1::HIS3MX6 wpl1::TRP1/wpl1::TRP1 PDS5/pds5::NatMX4 ECO1/eco1::KANMX4*	This study
HY10890	*MATa/MATalpha elg1::HIS3MX6/elg1::HIS3MX6 wpl1::TRP1/wpl1::TRP1 PDS5/pds5::NatMX4 RAD51/rad51::LEU2 SGS1/sgs1::KANMX4*	This study
HY11326	*MATa/MATalpha ELG1/elg1::HIS3MX6 WPL1/wpl1::TRP1 ECO1/eco1::KANMX4 SCC3/scc3-K404E-6HA::HPHNT1*	This study
HY11421	*MATa/MATalpha elg1::HIS3MX6/elg1::HIS3MX6 WPL1/wpl1::TRP1 PDS5/pds5::NatMX4 SCC3/scc3-K404E-6HA::HPHNT1*	This study
HY10996	*MATa/MATalpha ELG1/elg1::HPHMX6 CHL1/chl1::KANMX4*	This study
HY10997	*MATa/MATalpha ELG1/elg1::HPHMX6 CTF18/ctf18::TRP1*	This study
HY11097	*MATa/MATalpha wpl1::HIS3MX6/wpl1::HIS3MX6 elg1::HPHMX6/elg1::HPHMX6 CTF18/ctf18::TRP1 CHL1/chl1::KANMX4*	This study
HY11053	*MATa elg1::HIS3MX6 wpl1::TRP1 (natNT2)pGALS-3HA-PDS5*	This study
HY11081	*MATa elg1::HIS3MX6 wpl1::TRP1 (natNT2)pGALS-3HA-PDS5 ctf18::KANMX4*	This study
HY11083	*MATa elg1::HIS3MX6 wpl1::TRP1 (natNT2)pGALS-3HA-PDS5 chl1::KANMX4*	This study
HY11104	*MATa/MATalpha elg1::HIS3MX6/elg1::HIS3MX6 wpl1::TRP1/wpl1::TRP1 PDS5/pds5::NatMX4 SCC2/SCC2-6HA::HPHNT1*	This study
HY11121	*MATa/MATalpha elg1::HIS3MX6/elg1::HIS3MX6 wpl1::TRP1/wpl1::TRP1 PDS5/pds5::NatMX4 SCC2/scc2-pip-6HA::HPHNT1*	This study
HY11119	*MATa/MATalpha elg1::HIS3MX6/elg1::HIS3MX6 wpl1::TRP1/wpl1::TRP1 PDS5/pds5::NatMX4 SCC2/scc2-20-6HA::HPHNT1*	This study
HY9940	*MATa elg1::HIS3MX6 wpl1::TRP1*	This study
HY11212	*MATa elg1::HIS3MX6 wpl1::TRP1 SCC2-6HA::HPHNT1*	This study
HY11213	*MATa elg1::HIS3MX6 wpl1::TRP1 scc2-pip-6HA::HPHNT1*	This study
HY11214	*MATa elg1::HIS3MX6 wpl1::TRP1 scc2-20-6HA::HPHNT1*	This study
HY11218	*MATalpha elg1::HIS3MX6 wpl1::TRP1 SCC2-6HA::HPHNT1*	This study
HY11219	*MATalpha elg1::HIS3MX6 wpl1::TRP1 scc2-pip-6HA::HPHNT1*	This study
HY11220	*MATalpha elg1::HIS3MX6 wpl1::TRP1 scc2-20-6HA::HPHNT1*	This study
HY11289	*MATa/MATalpha elg1::HIS3MX6/elg1::HIS3MX6 wpl1::TRP1/wpl1::TRP1 CHL1/chl1::KANMX4 SCC2/SCC2-6HA::HPHNT1*	This study
HY11451	*MATa/MATalpha CHL1/chl1::KANMX4 SCC2/SCC2 (tSCC2::HPHNT1)*	This study
HY11452	*MATa/MATalpha CHL1/chl1::KANMX4 SCC2/scc2-pip::HPHNT1*	This study

HY11454	*MATa/MATalpha CTF18/ctf18::KANMX4 SCC2/scc2-pip::HPHNT1*	This study
HY11456	*MATa/MATalpha CTF19/ctf19::KlTRP1 SCC2/scc2-pip::HPHNT1*	This study
HY11459	*MATa/MATalpha elg1::HIS3MX6/elg1::HIS3MX6 wpl1::TRP1/wpl1::TRP1 PDS5/pds5::NatMX4 SCC2/SCC2 (tSCC2::HPHNT1)*	This study
HY11460	*MATa/MATalpha elg1::HIS3MX6/elg1::HIS3MX6 wpl1::TRP1/wpl1::TRP1 PDS5/pds5::NatMX4 SCC2/scc2-pip::HPHNT1*	This study
HY11575	*MATa/MATalpha MRC1/mrc1::KANMX4 SCC2/scc2-pip::HPHNT1*	This study
HY11602	*MATa/MATalpha CTF4/ctf4::TRP1 SCC2/scc2-pip::HPHNT1*	This study
HY11603	*MATa/MATalpha CSM3/csm3::HIS3MX6 SCC2/scc2-pip::HPHNT1*	This study
HY11604	*MATa/MATalpha TOF1/tof1::KANMX6 SCC2/scc2-pip::HPHNT1*	This study
HY9980	*MATalpha elg1::HIS3MX6 wpl1::TRP1 pds5::NatMX4*	This study
HY11379	*MATa elg1::HIS3MX6 wpl1::TRP1 SCC2 (tSCC2::HPHNT1)*	This study
HY11382	*MATa elg1::HIS3MX6 wpl1::TRP1 scc2-pip::HPHNT1*	This study
HY11461	*MATa elg1::HIS3MX6 wpl1::TRP1 SCC2 (tSCC2::HPHNT1) pds5::NatMX4*	This study
HY11463	*MATa elg1::HIS3MX6 wpl1::TRP1 scc2-pip::HPHNT1 pds5::NatMX4*	This study
HY11377	*MATa SCC2 (tSCC2::HPHNT1)*	This study
HY11380	*MATa scc2-pip::HPHNT1*	This study
HY2192	*MATalpha chl1::KANMX4*	Lab collection
HY11309	*MATalpha ctf18::KANMX4*	This study
HY11301	*MATalpha ctf19::KlTRP1*	This study
HY11427	*MATa SCC2 (tSCC2::HPHNT1) chl1::KANMX4*	This study
HY11429	*MATa SCC2 (tSCC2::HPHNT1) ctf18::KANMX4*	This study
HY11435	*MATa scc2-pip::HPHNT1 chl1::KANMX4*	This study
HY11437	*MATa scc2-pip::HPHNT1 ctf18::KANMX4*	This study
HY11630	*MATa scc2-pip::HPHNT1 ctf4::TRP1*	This study
HY11632	*MATa scc2-pip::HPHNT1 csm3::HIS3MX6*	This study
HY11634	*MATa scc2-pip::HPHNT1 tof1::KANMX6*	This study
HY11580	*MATa scc2-pip::HPHNT1 mrc1::KANMX4*	This study
HY6823	*MATalpha mrc1::KANMX4*	This study
HY1613	*MATalpha ctf4::TRP1*	Lab collection
HY2844	*MATalpha tof1::KANMX6*	Lab collection
HY4479	*MATa csm3::HIS3MX6*	Lab collection
HY11479	*MATa his3-11,15::HIS3tetR-GFP (single integrant), ura3::3xURA3tetO112 SCC2 (tSCC2::HPHNT1)*	This study
HY11480	*MATa his3-11,15::HIS3tetR-GFP (single integrant), ura3::3xURA3tetO112 SCC2 (tSCC2::HPHNT1) chl1::TRP1*	This study
HY11481	*MATa his3-11,15::HIS3tetR-GFP (single integrant), ura3::3xURA3tetO112 scc2-pip::HPHNT1*	This study
HY11483	*MATa his3-11,15::HIS3tetR-GFP (single integrant), ura3::3xURA3tetO112 scc2-pip::HPHNT1 chl1::TRP1*	This study
HY11548	*MATa his3-11,15::HIS3tetR-GFP (single integrant), ura3::3xURA3tetO112 SCC2 (tSCC2::HPHNT1) ctf18::TRP1*	This study
HY11549	*MATa his3-11,15::HIS3tetR-GFP (single integrant), ura3::3xURA3tetO112 scc2-pip::HPHNT1 ctf18::NatNT2*	This study
HY11489	*MATa/MATalpha SCC2 (tSCC2::HPHNT1)/SCC2*	This study

	(tSCC2::HPHNT1) CTF18/ctf18::KANMX4 CTF19/ctf19::KlTRP1	
HY11490	*MATa/MATalpha scc2-pip::HPHNT1/scc2-pip::HPHNT1 CTF18/ctf18::KANMX4 CTF19/ctf19::KlTRP1*	This study
HY11431	*MATa SCC2 (tSCC2::HPHNT1) ctf19::KlTRP1*	This study
HY11439	*MATa scc2-pip::HPHNT1 ctf19::KlTRP1*	This study
HY11509	*MATa SCC2 (tSCC2::HPHNT1) ctf18::KANMX4 ctf19::KlTRP1*	This study
HY11511	*MATa scc2-pip::HPHNT1 ctf18::KANMX4 ctf19::KlTRP1*	This study
HY12024	*MATa/MATalpha SCC2/scc2-E822K::HPHNT1 SCC4/(KANMX4)pGALS-SCC4*	This study
HY12025	*MATa/MATalpha SCC2/scc2-E822K-pip::HPHNT1 SCC4/(KANMX4)pGALS-SCC4*	This study
HY12037	*MATa/MATalpha SCC2/scc2-E822K-pol32PIP::NatNT2 SCC4/(KANMX4)pGALS-SCC4*	This study
HY12038	*MATa/MATalpha SCC2/scc2-E822K-pol32pip::NatNT2 SCC4/(KANMX4)pGALS-SCC4*	This study
HY12039	*MATa/MATalpha SCC2/scc2-E822K-17aa::NatNT2 SCC4/(KANMX4)pGALS-SCC4*	This study
HY12040	*MATa/MATalpha SCC2/scc2-E822K-pip-pol32PIP::NatNT2 SCC4/(KANMX4)pGALS-SCC4*	This study
HY11750	*MATalpha scc2-E822K::HPHNT1 (KANMX4)pGALS-SCC4*	This study
HY11746	*MATalpha scc2-E822K-pip::HPHNT1 (KANMX4)pGALS-SCC4*	This study
HY11999	*MATa (natNT2)pADH1-7His8FLAG-scc2-E822K::HPHNT1*	This study
HY11971	*MATa (natNT2)pADH1-7His8FLAG-scc2-E822K-pip::HPHNT1*	This study
HY12139	*MATa pSCC2-7His8FLAG-scc2-E822K::natNT2*	This study
HY12134	*MATa pSCC2-7His8FLAG-scc2-E822K-pip::natNT2*	This study
HY12136	*MATa pSCC2-7His8FLAG-scc2-E822K-pol32PIP::NatNT2*	This study
HY12137	*MATa pSCC2-7His8FLAG-scc2-E822K-pol32pip::NatNT2*	This study
HY11937	*MATalpha (natNT2)pADH1-7His10FLAG-scc2-E822K::HPHNT1 (KANMX4)pGALS-SCC4*	This study
HY12047	*MATa POL30-3MYC::KANMX4*	This study
HY12089	*MATa/MATalpha POL30/POL30-3MYC::KANMX4 SCC2/scc2-E822K::HPHNT1 SCC4/scc4::NatNT2*	This study
HY12090	*MATa scc2-E822K::HPHNT1 scc4::NatNT2*	This study
HY12093	*MATalpha scc2-E822K::HPHNT1 scc4::NatNT2*	This study
HY12095	*MATalpha POL30-3MYC::KANMX4 scc2-E822K::HPHNT1 scc4::NatNT2*	This study
HY12144	*MATa/MATalpha pol30-6/POL30-3MYC::KANMX4 scc2-E822K::HPHNT1/scc2-E822K::HPHNT1 SCC4/scc4::NatNT2*	This study
HY12150	*MATa/MATalpha pol30-79/POL30-3MYC::KANMX4 scc2-E822K::HPHNT1/scc2-E822K::HPHNT1 SCC4/scc4::NatNT2*	This study
HY12147	*MATa/MATalpha pol30-8/POL30-3MYC::KANMX4 scc2-E822K::HPHNT1/scc2-E822K::HPHNT1 SCC4/scc4::NatNT2*	This study
HY12151	*MATa pol30-79 scc2-E822K::HPHNT1 scc4::NatNT2*	This study
HY12048	*MATa (natNT2)pADH1-7His8FLAG-scc2-E822K::HPHNT1 POL30-3MYC::KANMX4*	This study
HY12050	*MATa (natNT2)pADH1-7His8FLAG-scc2-E822K-pip::HPHNT1 POL30-3MYC::KANMX4*	This study
HY11980	*MATa/MATalpha SCC2/scc2-E822K::HPHNT1 SCC4/(KANMX4)pGALS-SCC4 CTF18/ctf18::TRP1*	This study
HY12061	*MATa/MATalpha SCC2/scc2-E822K::HPHNT1 SCC4/(KANMX4)pGALS-SCC4 CHL1/chl1::URA3*	This study
HY11979	*MATa/MATalpha SCC4/(KANMX4)pGALS-SCC4 CTF18/ctf18::TRP1 SCC2/(natNT2)pADH1-7His8FLAG-scc2-*	This study

	E822K::HPHNT1	
HY12071	*MATa/MATalpha SCC4/(KANMX4)pGALS-SCC4 CHL1/chl1::URA3 SCC2/(natNT2)pADH1-7His8FLAG-scc2-E822K::HPHNT1*	This study
HY11964	*MATa/MATalpha SCC4/(KANMX4)pGALS-SCC4 SCC2/(natNT2)pADH1-7His8FLAG-scc2-E822K-pip::HPHNT1*	This study
HY12026	*MATa/MATalpha SCC4/(KANMX4)pGALS-SCC4 CTF18/ctf18::TRP1 SCC2/(natNT2)pADH1-7His8FLAG-scc2-E822K-pip::HPHNT1*	This study
HY12062	*MATa/MATalpha SCC2/scc2-E822K::HPHNT1 SCC4/(KANMX4)pGALS-SCC4 CHL1/chl1-K48R-3HA::LEU2*	This study
HY12072	*MATa/MATalpha SCC4/(KANMX4)pGALS-SCC4 CHL1/chl1-K48R-3HA::LEU2 SCC2/(natNT2)pADH1-7His8FLAG-scc2-E822K::HPHNT1*	This study
HY12027	*MATa/MATalpha CTF18/ctf18::TRP1 CHL1/chl1::KANMX4 SCC2/(natNT2)pADH1-7His8FLAG-scc2-E822K::HPHNT1*	This study
HY12105	*MATa/MATalpha CTF18/ctf18::TRP1 CHL1/chl1-K48R-3HA::LEU2 (natNT2)pADH1-7His8FLAG-scc2-E822K::HPHNT1/(natNT2)pADH1-7His8FLAG-scc2-E822K::HPHNT1*	This study
HY12104	*MATa/MATalpha CTF18/ctf18::TRP1 CHL1/chl1-K48R-3HA::LEU2 (natNT2)pADH1-7His8FLAG-scc2-E822K::HPHNT1/(natNT2)pADH1-7His8FLAG-scc2-E822K::HPHNT1 (KANMX4)pGALS-SCC4/(KANMX4)pGALS-SCC4*	This study
HY12153	*MATa/MATalpha SCC2/pSCC2-7His8FLAG-scc2-E822K::natNT2 SCC4/(KANMX4)pGALS-SCC4*	This study
HY12154	*MATa/MATalpha SCC2/pSCC2-7His8FLAG-scc2-E822K-pip::natNT2 SCC4/(KANMX4)pGALS-SCC4*	This study
HY12155	*MATa/MATalpha SCC2/pSCC2-7His8FLAG-scc2-E822K-pol32PIP::NatNT2 SCC4/(KANMX4)pGALS-SCC4*	This study
HY12156	*MATa/MATalpha SCC2/pSCC2-7His8FLAG-scc2-E822K-pol32pip::NatNT2 SCC4/(KANMX4)pGALS-SCC4*	This study
HY12171	*MATalpha pSCC2-7His8FLAG-scc2-E822K::natNT2*	This study
HY12172	*MATalpha pSCC2-7His8FLAG-scc2-E822K-pip::natNT2*	This study
HY12173	*MATalpha pSCC2-7His8FLAG-scc2-E822K-pol32PIP::NatNT2*	This study
HY12174	*MATalpha pSCC2-7His8FLAG-scc2-E822K-pol32pip::NatNT2*	This study
HY11952	*MATalpha (natNT2)pADH1-7His8FLAG-scc2-E822K::HPHNT1 (KANMX4)pGALS-SCC4*	This study
HY11972	*MATa (natNT2)pADH1-7His8FLAG-scc2-E822K-pip::HPHNT1 (KANMX4)pGALS-SCC4*	This study
HY12450	*MATa/MATalpha SCC1/(TRP1)pGAL1-SCC1 ELG1/elg1::HPHMX6 WPL1/wpl1::HIS3MX6 PDS5/pds5::NatMX4*	This study
HY12492	*MATa (TRP1)pGAL1-SCC1*	This study
HY12493	*MATa (TRP1)pGAL1-SCC1 elg1::HPHMX6*	This study
HY12494	*MATa (TRP1)pGAL1-SCC1 elg1::HPHMX6 pds5::NatMX4*	This study
HY12682	*MATa/MATalpha elg1::HIS3MX6/elg1::HIS3MX6 wpl1::TRP1/wpl1::TRP1 PDS5/pds5::NatMX4 SRS2/srs2::KANMX4 RAD51/rad51::LEU2*	This study
HY12685	*MATa/MATalpha elg1::HIS3MX6/elg1::HIS3MX6 wpl1::TRP1/wpl1::TRP1 PDS5/pds5::NatMX4 SRS2/srs2-ΔC138::KANMX4 RAD51/rad51::LEU2*	This study
HY12956	*MATalpha elg1::HIS3MX6 wpl1::TRP1 pds5::NatMX4 (KANMX4)pGAL1-3HA-RAD51*	This study
HY12957	*MATalpha elg1::HIS3MX6 wpl1::TRP1 pds5::NatMX4 (KANMX4)pGAL1-3HA-RAD51 srs2-ΔC138::KANMX4*	This study

HY12960	*MATa elg1::HIS3MX6 wpl1::TRP1 pds5::NatMX4 (KANMX4)pGAL1-3HA-RAD51 srs2::KANMX4*	This study
HY12924	*MATa (natNT2)pADH1-3HA-scc2-Q1475TAG::HPHNT1 POL30-3MYC::KANMX4* [pLH157 (*TRP1 EcTyrRS EctRNACUA)*]	This study
HY12925	*MATa (natNT2)pADH1-3HA-scc2-T1493TAG::HPHNT1 POL30-3MYC::KANMX4* [pLH157 (*TRP1 EcTyrRS EctRNACUA)*]	This study

All strains are isogenic to W303 background.

Supplementary Table 2. Cell lines used in this study.

Cell Type	Code	Strain Name	Genotype	Marker	Reference
TK6	R316	WT	TSCER2 +*TIR1*	Puro	(Hoa et al., 2015)
	#100	*tp53*	TSCER2 *TP53*$^{-/-}$	-	This study. Gift from H. Sasanuma
DT40	I008	WT	WT CL18	-	(Buerstedde et al., 1991)
	R448	WT +*3HA-NIPBL*	WT +*TIR1-9Myc*	Neo	This study
	R459	*chr.w*	*Chr.W*<br>*+TIR1-9Myc*	-<br>Neo	This study
	R464<br>R465	*chr.w*<br>*nipbl-ΔC-EGFP*	*Chr.W*<br>*NIPBL*$^{2588\text{-}EGFP}$<br>*+TIR1-9Myc*	-<br>Eco<br>Neo	This study
	R530	*chr.w*<br>*NIPBL-EGFP*	*Chr.W*<br>*NIPBL*^{EGFP}<br>*+TIR1-9Myc*	-<br>Eco<br>Neo	This study
	R522	*chr.w*<br>*nipbl-ΔC-EGFP*<br>*ctf18*	*Chr.W*<br>*NIPBL*$^{2588\text{-}EGFP}$<br>*CTF18*$^{-/-}$<br>*+TIR1-9Myc*	-<br>Eco<br>His/His<br>Neo	This study
	R524	*chr.w*<br>*ddx11*	*Chr.W*<br>*DDX11*$^{-/-}$<br>*+TIR1-9Myc*	-<br>Puro/Puro<br>Neo	This study
	R526	*chr.w*<br>*ctf18*	*Chr.W*<br>*CTF18*$^{-/-}$<br>*+TIR1-9Myc*	-<br>His/His<br>Neo	This study
	R535	*chr.w*<br>*nipbl-ΔC-EGFP*<br>*ddx11-aid*	*Chr.W*<br>*NIPBL*$^{2588\text{-}EGFP}$<br>*DDX11*$^{3mAID6FLAG/3mAID6FLAG}$<br>*+TIR1-9Myc*	-<br>Eco<br>Bleo/Bleo<br>Neo	This study
	R537	*chr.w*<br>*ctf18*<br>*ddx11-aid*	*Chr.W*<br>*CTF18*$^{-/-}$<br>*DDX11*$^{3mAID6FLAG/3mAID6FLAG}$<br>*+TIR1-9Myc*	-<br>His/His<br>Bleo/Bleo<br>Neo	This study
	T392	*miniChr.2-mCherry*	*Telomere-EGFR*<br>*Telomere-TPK1-mCherry*	Puro<br>His	This study
	T396	*miniChr.2-mCherry*<br>*chr.w*	*Telomere-EGFR*<br>*Telomere-TPK1-mCherry*<br>*Chr.W*	Puro<br>His	This study
	T397	*miniChr.2-mCherry*<br>*chr.w*<br>*nipbl-ΔC-EGFP*	*Telomere-EGFR*<br>*Telomere-TPK1-mCherry*<br>*Chr.W*<br>*NIPBL*$^{2588\text{-}EGFP}$	Puro<br>His<br>-<br>Eco	This study
