# Supplementary material for: PCNA recruits cohesin loader Scc2 to ensure sister chromatid cohesion
Source: Nat Struct Mol Biol. 2023 Aug 17;30(9):1286–94. doi: 10.1038/s41594-023-01064-x (PMC10497406; doi:10.1038/s41594-023-01064-x)
Supplement: Supplementary file 2 — Reporting Summary [file 41594_2023_1064_MOESM2_ESM.pdf]

nature portfolio

Corresponding author(s): Dana Branzei and Ivan Psakhye

Last updated by author(s): May 2, 2023

Reporting Summary

Nature Portfolio wishes to improve the reproducibility of the work that we publish. This form provides structure for consistency and transparency in reporting. For further information on Nature Portfolio policies, see our Editorial Policies and the Editorial Policy Checklist.

Statistics

For all statistical analyses, confirm that the following items are present in the figure legend, table legend, main text, or Methods section.

n/a	Confirmed	
☐	☒	The exact sample size (*n*) for each experimental group/condition, given as a discrete number and unit of measurement
☐	☒	A statement on whether measurements were taken from distinct samples or whether the same sample was measured repeatedly
☐	☒	The statistical test(s) used AND whether they are one- or two-sided<br>*Only common tests should be described solely by name; describe more complex techniques in the Methods section.*
☒	☐	A description of all covariates tested
☒	☐	A description of any assumptions or corrections, such as tests of normality and adjustment for multiple comparisons
☐	☒	A full description of the statistical parameters including central tendency (e.g. means) or other basic estimates (e.g. regression coefficient) AND variation (e.g. standard deviation) or associated estimates of uncertainty (e.g. confidence intervals)
☐	☒	For null hypothesis testing, the test statistic (e.g. *F*, *t*, *r*) with confidence intervals, effect sizes, degrees of freedom and *P* value noted<br>*Give P values as exact values whenever suitable.*
☒	☐	For Bayesian analysis, information on the choice of priors and Markov chain Monte Carlo settings
☒	☐	For hierarchical and complex designs, identification of the appropriate level for tests and full reporting of outcomes
☒	☐	Estimates of effect sizes (e.g. Cohen's *d*, Pearson's *r*), indicating how they were calculated

Our web collection on statistics for biologists contains articles on many of the points above.

Software and code

Policy information about availability of computer code

Data collection	BIORAD Image Lab Version 5.2.1 for Western Blot acquisition<br>BIORAD ChemiDoc Touch Imaging System for Western Blot and Slot Blot<br>BD Accuri C6 Plus for FACS sample collection<br>Roche LightCycler 96 version 1.1.0.1320 for qPCR<br>DeltaVision microscope (Applied Precision) for premature sister chromatid separation assay in budding yeast<br>Nikon NIS-Elements for cohesion assay in DT40 cells<br>SkanIt 6.1 for CellTiter-Glo assay
Data analysis	BIORAD Image Lab Version 5.2.1 for quantification of Western Blots<br>BD Accuri C6 Plus for FACS analysis<br>GraphPad Prism Version 9.0.2 (161) for ChIP-qPCR analysis<br>ImageJ 1.49v for image analysis of premature sister chromatid separation assay in budding yeast<br>Google DeepMind alphaFold2 and alphaFold-Multimer, v2.3.2<br>PyMOL molecular graphics system, v1.7.4.5 Schrödinger, LLC

For manuscripts utilizing custom algorithms or software that are central to the research but not yet described in published literature, software must be made available to editors and reviewers. We strongly encourage code deposition in a community repository (e.g. GitHub). See the Nature Portfolio guidelines for submitting code & software for further information.

Data

Policy information about availability of data

All manuscripts must include a data availability statement. This statement should provide the following information, where applicable:

- Accession codes, unique identifiers, or web links for publicly available datasets
- A description of any restrictions on data availability
- For clinical datasets or third party data, please ensure that the statement adheres to our policy

The authors declare that the data supporting the findings of this study are available within the article. Source data are provided with this paper, and archived at the IFOM ETS, the AIRC Institute of Molecular Oncology, or the Department of Chemistry at Tokyo Metropolitan University. The following publicly available datasets were used in the study: PDB identifiers 6ZZ6, 6WGE, and 6YUF.

Human research participants

Policy information about studies involving human research participants and Sex and Gender in Research.

Reporting on sex and gender	NA
Population characteristics	NA
Recruitment	NA
Ethics oversight	NA

Note that full information on the approval of the study protocol must also be provided in the manuscript.

Field-specific reporting

Please select the one below that is the best fit for your research. If you are not sure, read the appropriate sections before making your selection.

☒ Life sciences ☐ Behavioural & social sciences ☐ Ecological, evolutionary & environmental sciences

For a reference copy of the document with all sections, see nature.com/documents/nr-reporting-summary-flat.pdf

Life sciences study design

All studies must disclose on these points even when the disclosure is negative.

Sample size	No statistical methods were used to predetermine sample size, as this study did not include animal models or human participants. Sample size was determined based on the established standards in the field and experiments to obtain statistical significance and reproducibility. We used 10 000 cells for each flow cytometry analysis point (Abe et al., Sci Rep, 2021), 10 million cells for each ChIP sample (Psakhye et al., Mol Cell, 2019), over 100 nuclei analysis for cohesion assays in DT40 cells (Kawasumi et al, Genes Dev, 2021), over 200 cells per each yeast strain for premature sister chromatid separation assay in budding yeast (Michaelis et al., Cell, 1997).
Data exclusions	No data were excluded.
Replication	All experimental findings were reliably reproduced as indicated in the figure legends. All experiments were repeated at least 2 times to ensure reproducibility. We have not experienced cases of non-reproducible data in this study. At least three biological replicates were included for ChIP-qPCR and cohesion assays. The mean values of at least 3 experiments were calculated and shown with corresponding SD or SEM. Statistical analyses were performed using unpaired two-tailed Student's t-test.
Randomization	No randomization was done because this study did not involve animals or human participants. Samples were organized into groups based on treatment and genotype. Appropriate controls were included in all experiments.
Blinding	Before each experiment, the strains that were used were given numbers instead of the genotype or condition and the numbers were then connected to the yeast strains or cell line genotype only after analysis.

Reporting for specific materials, systems and methods

We require information from authors about some types of materials, experimental systems and methods used in many studies. Here, indicate whether each material, system or method listed is relevant to your study. If you are not sure if a list item applies to your research, read the appropriate section before selecting a response.

Materials & experimental systems

n/a	Involved in the study
☐	☒ Antibodies
☐	☒ Eukaryotic cell lines
☒	☐ Palaeontology and archaeology
☒	☐ Animals and other organisms
☒	☐ Clinical data
☒	☐ Dual use research of concern

Methods

n/a	Involved in the study
☒	☐ ChIP-seq
☐	☒ Flow cytometry
☒	☐ MRI-based neuroimaging

Antibodies

Antibodies used	Mouse monoclonal anti-FLAG antibody (1:2000, clone M2; F3165) was purchased from Sigma-Aldrich. Mouse monoclonal anti-Pgk1 antibody (1:2000, clone 22C5D8; cat # 459250) was obtained from Thermo Fisher Scientific. Mouse monoclonal anti-HA (1:2000, clone F-7; sc-7392) and anti-PCNA (1:2000, clone F-2; sc-25280) antibodies were from Santa Cruz Biotechnology, as well as normal mouse IgG. Rabbit polyclonal anti-Pol30 antibody (1:2000; GTX64144) was purchased from Gene Tex. Mouse monoclonal anti-c-MYC antibody (1:2000, clone 9E10) and rabbit polyclonal anti-GST antibody (1:2000) were produced in house. Rabbit polyclonal anti-Histone H4 antibody (1:2000; ab7311) was obtained from Abcam. Mouse monoclonal anti-acetyl-Smc3 antibody (1:2000) was a gift from Katsuhiko Shirahige (Borges et al., 2010). Rabbit polyclonal anti-Mcm2-7 antibody (1:5000; UM185) was a gift from Stephen P. Bell (Bowers et al., 2004). anti-NIPBL antibody (1:1000; A301-779A) was from Bethyl Laboratories. anti-SMC3 antibody (1:1000) was a gift from Ana Losada. anti-BrdU antibody (1:500; 347580) was from BD Biosciences. anti-GAPDH antibody (1:10000; sc-47724), anti-GFP antibody (1:500; sc-9996), anti-MCM7 antibody (1:500; sc-9966), anti-NIPBL (1:200; sc-374625) were purchased from from Santa Cruz Biotechnology. anti-HA antibody (1:1000; 11867423001) was from Roche. anti-MAU2 antibody (1:1000; ab183033) was purchased from Abcam. anti-miniAID antibody (1:1000; M214-3) was from MBL. Anti-rabbit IgG (7074S) and anti-mouse IgG (7076S), HRP-linked antibodies (1:5000) were purchased from Cell Signaling Technology.
Validation	All antibodies in this study were used for Western Blot analysis in S. cerevisiae yeast, human TK6 and chicken DT40 samples and the bands for the respective proteins corresponded with the expected size. The application (Western Blot) and species were indicated on the manufacturers websites.<br>We have validated anti-HA antibody in Western Blot by using an S. cerevisiae yeast strain without any tag to confirm specificity. The Pgk1 antibody is commonly used as a loading control in many publications (Gay et al., 2018).

Eukaryotic cell lines

Policy information about cell lines and Sex and Gender in Research

Cell line source(s)	Budding yeast Saccharomyces cerevisiae strains (W303 background), chicken DT40, and human TK6 (Japanese Collection of Research Bioresources Cell Bank; https://cellbank.nibiohn.go.jp) cell lines.
Authentication	The yeast strains and chicken DT40 cell lines were confirmed with resistance markers, PCR, sequencing, western blotting wherever relevant. The human TK6 cell lines used were not authenticated.
Mycoplasma contamination	All cell lines used were tested negative for mycoplasma contamination.
Commonly misidentified lines<br>(See ICLAC register)	No commonly misidentified cell lines were used in the study.

Flow Cytometry

Plots

Confirm that:

☒ The axis labels state the marker and fluorochrome used (e.g. CD4-FITC).

☒ The axis scales are clearly visible. Include numbers along axes only for bottom left plot of group (a 'group' is an analysis of identical markers).

☒ All plots are contour plots with outliers or pseudocolor plots.

☒ A numerical value for number of cells or percentage (with statistics) is provided.

Methodology

Sample preparation	Exponentially growing DT40 cells were analyzed by flow cytometry without any preparation procedures.
Instrument	BD Accuri C6 Plus Flow Cytometry
Software	BD Accuri C6 Plus

March 2021

Cell population abundance	About 70% were living cells, which were tested for mCherry (FL3) signal.
Gating strategy	Samples were gated on SSC-A and FSC-A to exclude dead cells and debris. Then living cells (P1) were gated on SSC-A and FL3 to determine the percentage of mCherry negative cells (P2). 10000 of living cells per sample were analysed.

☒ Tick this box to confirm that a figure exemplifying the gating strategy is provided in the Supplementary Information.
