# Supplementary figures and images for: PCNA recruits cohesin loader Scc2 to ensure sister chromatid cohesion
Source: Nat Struct Mol Biol. 2023 Aug 17;30(9):1286–94. doi: 10.1038/s41594-023-01064-x (PMC10497406; doi:10.1038/s41594-023-01064-x)

Raw Data Figure 2

c

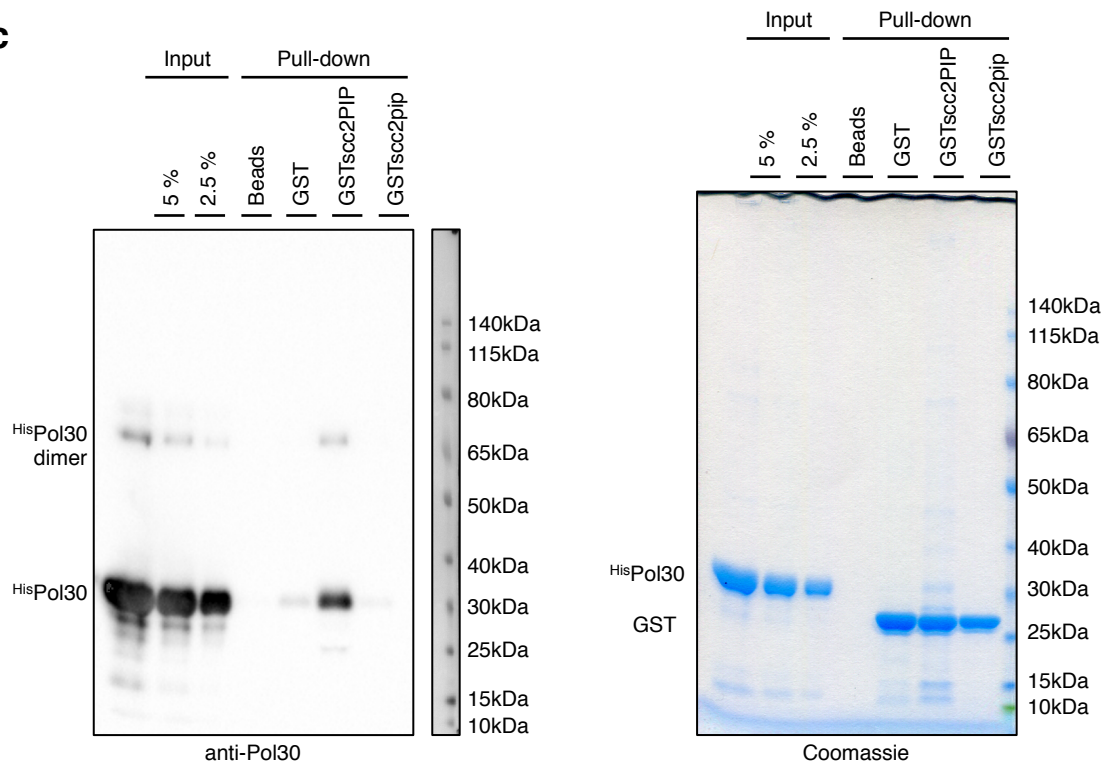

d

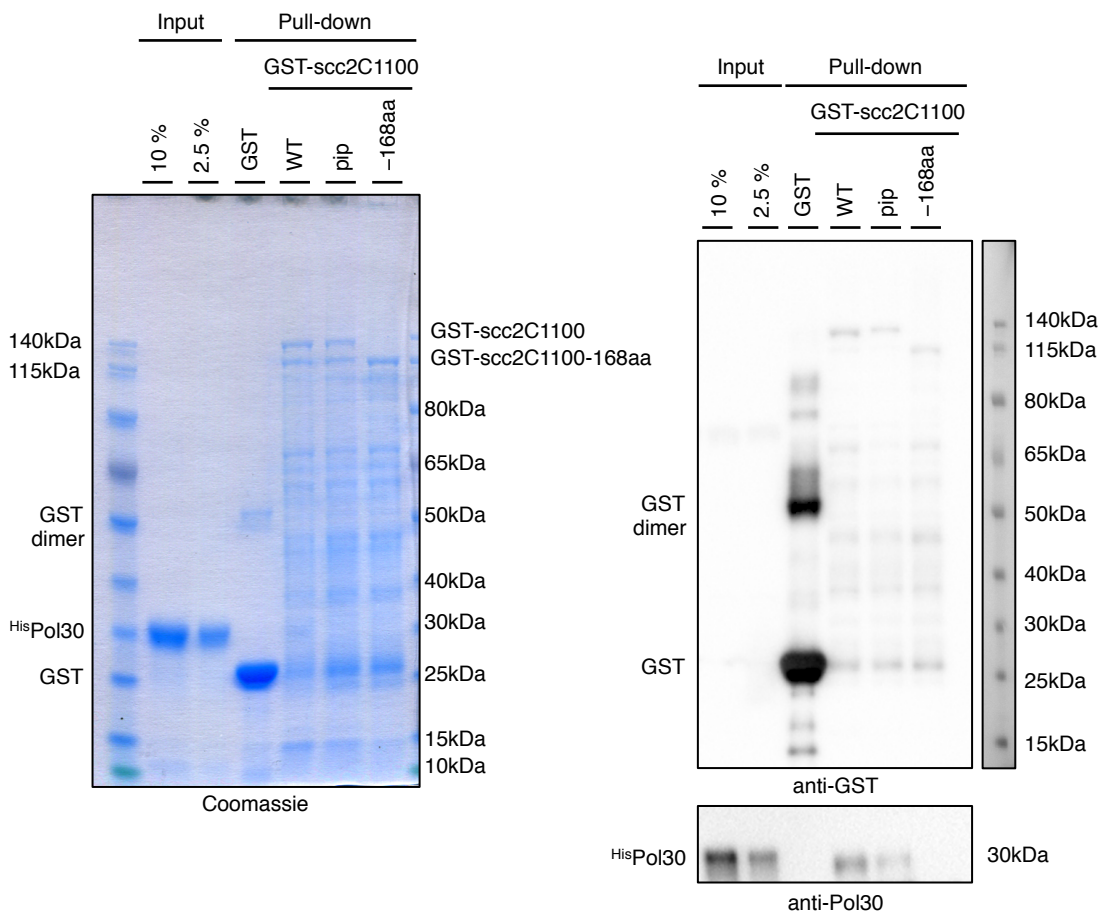

Supplement: Source Data Fig. 2 — Unprocessed western blots and gels. [file 41594_2023_1064_MOESM4_ESM.pdf]

Raw Data Figure 4

a

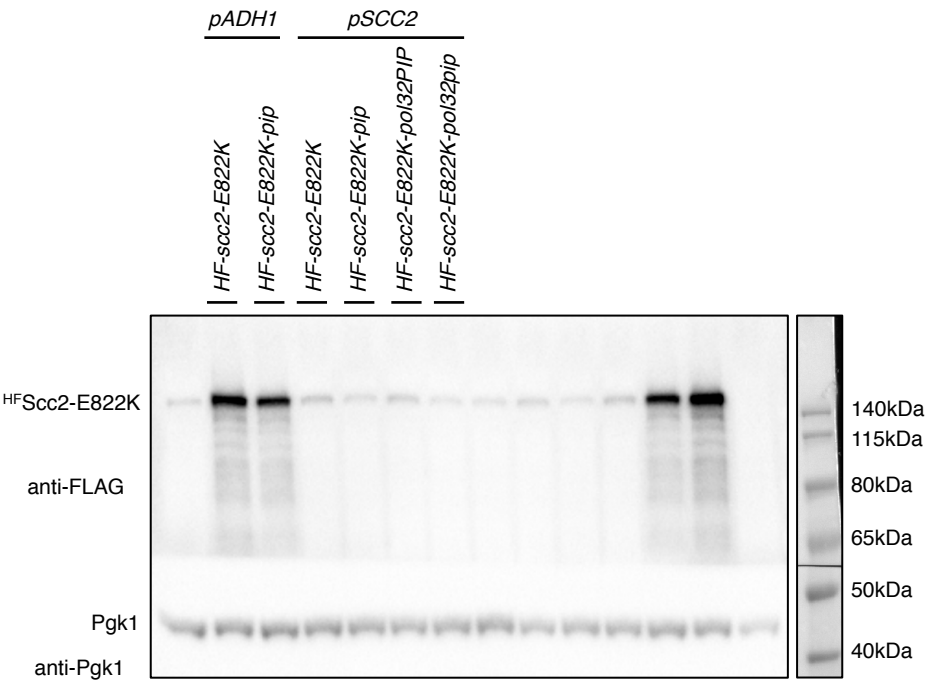

b

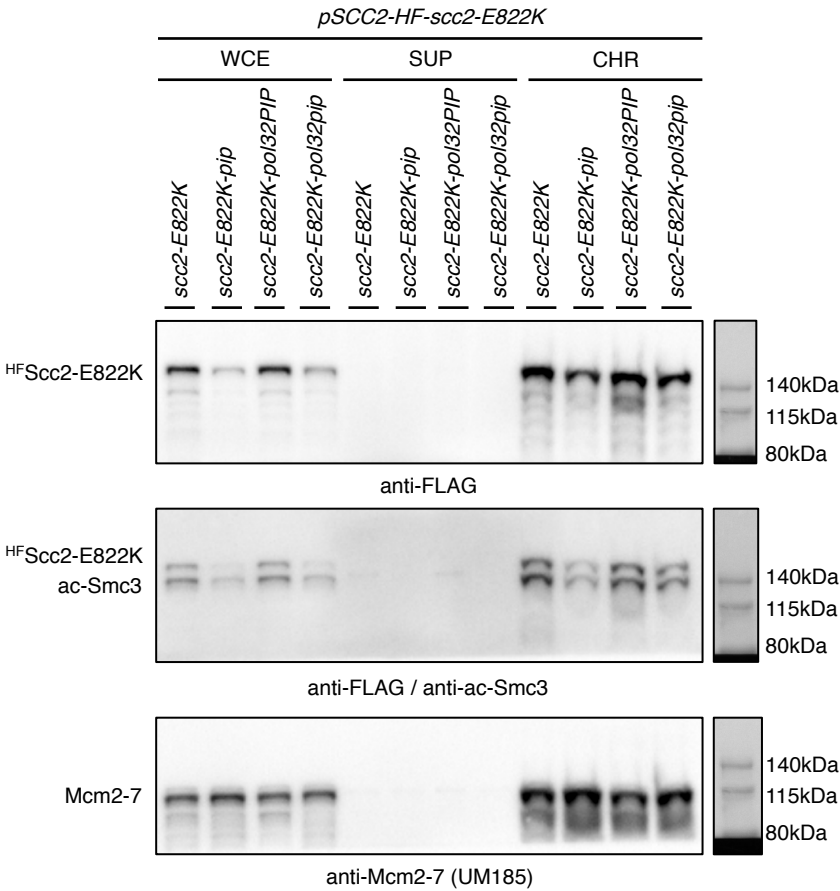

Raw Data Figure 4

b

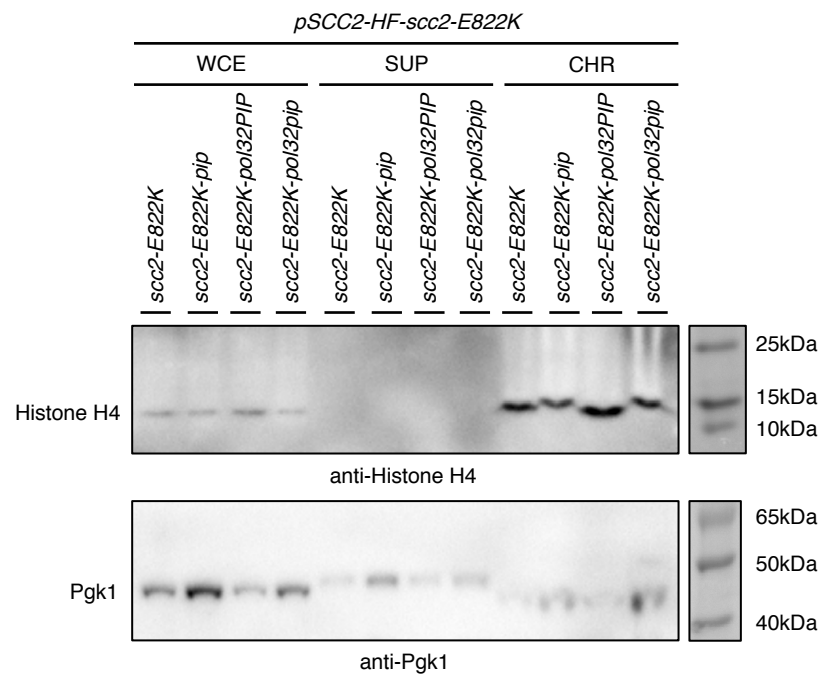

Supplement: Source Data Fig. 4 — Unprocessed western blots. [file 41594_2023_1064_MOESM5_ESM.pdf]

Raw Data Figure 5

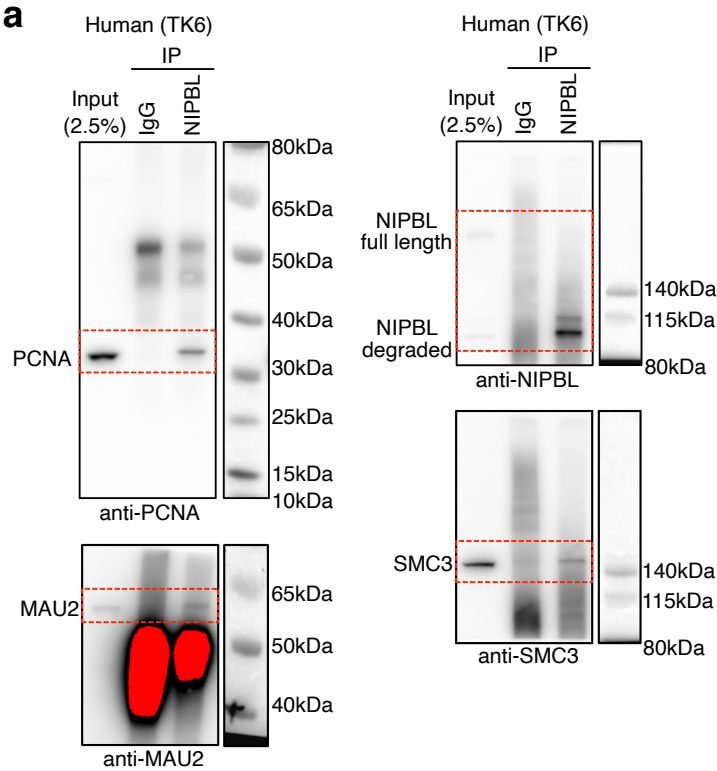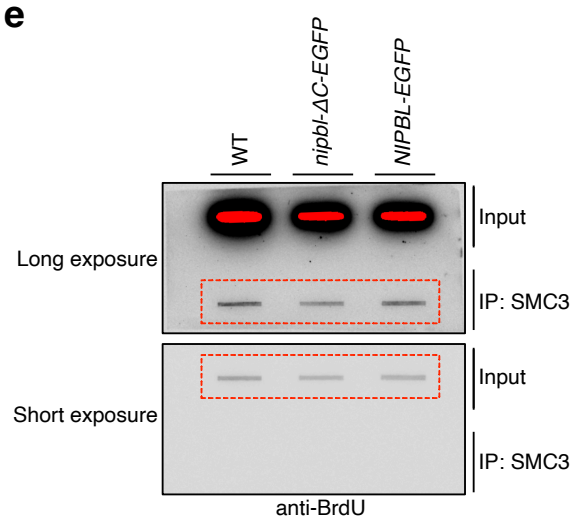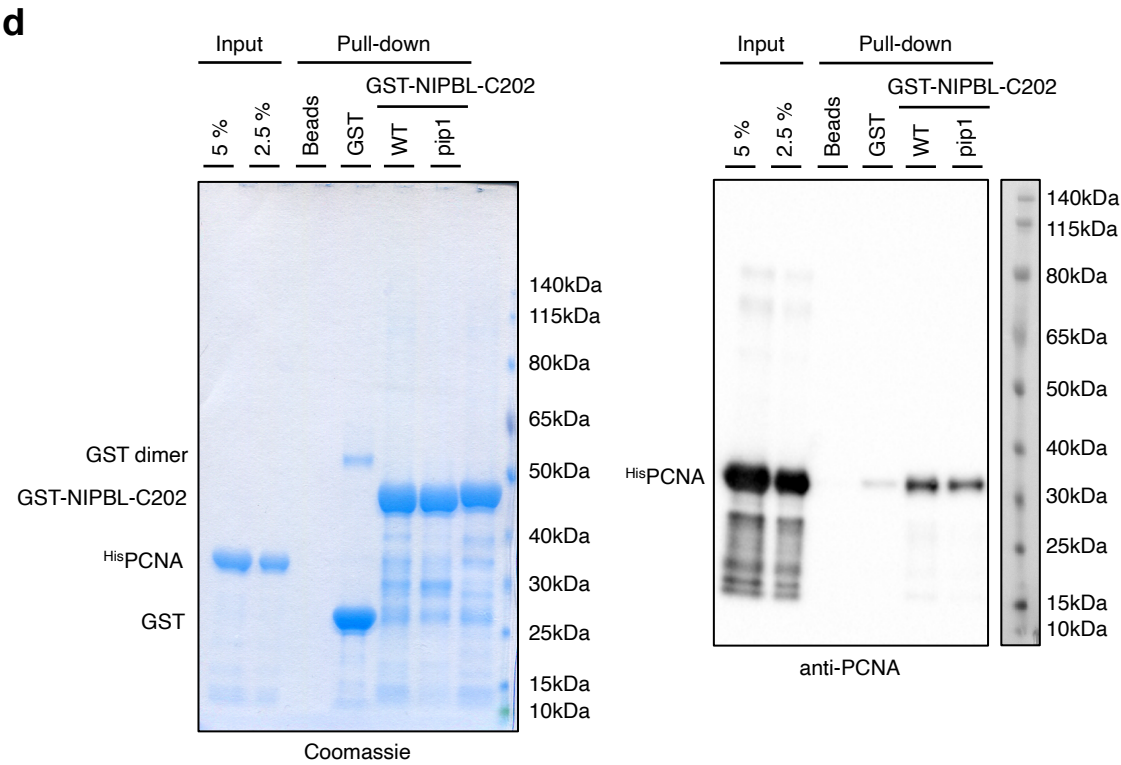

Supplement: Source Data Fig. 5 — Unprocessed western blots and gels. [file 41594_2023_1064_MOESM6_ESM.pdf]

Raw Data Extended Data Figure 1

d

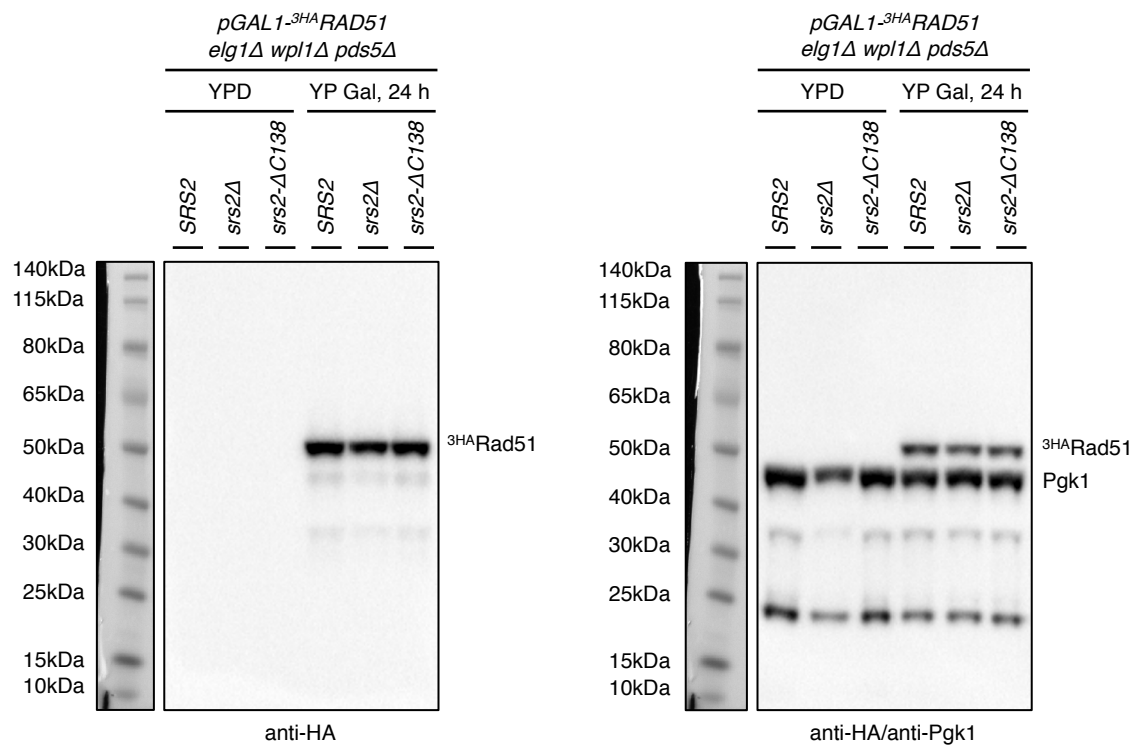

Supplement: Source Data Extended Data Fig. 1 — Unprocessed western blots. [file 41594_2023_1064_MOESM11_ESM.pdf]

Raw Data Extended Data Figure 2

b

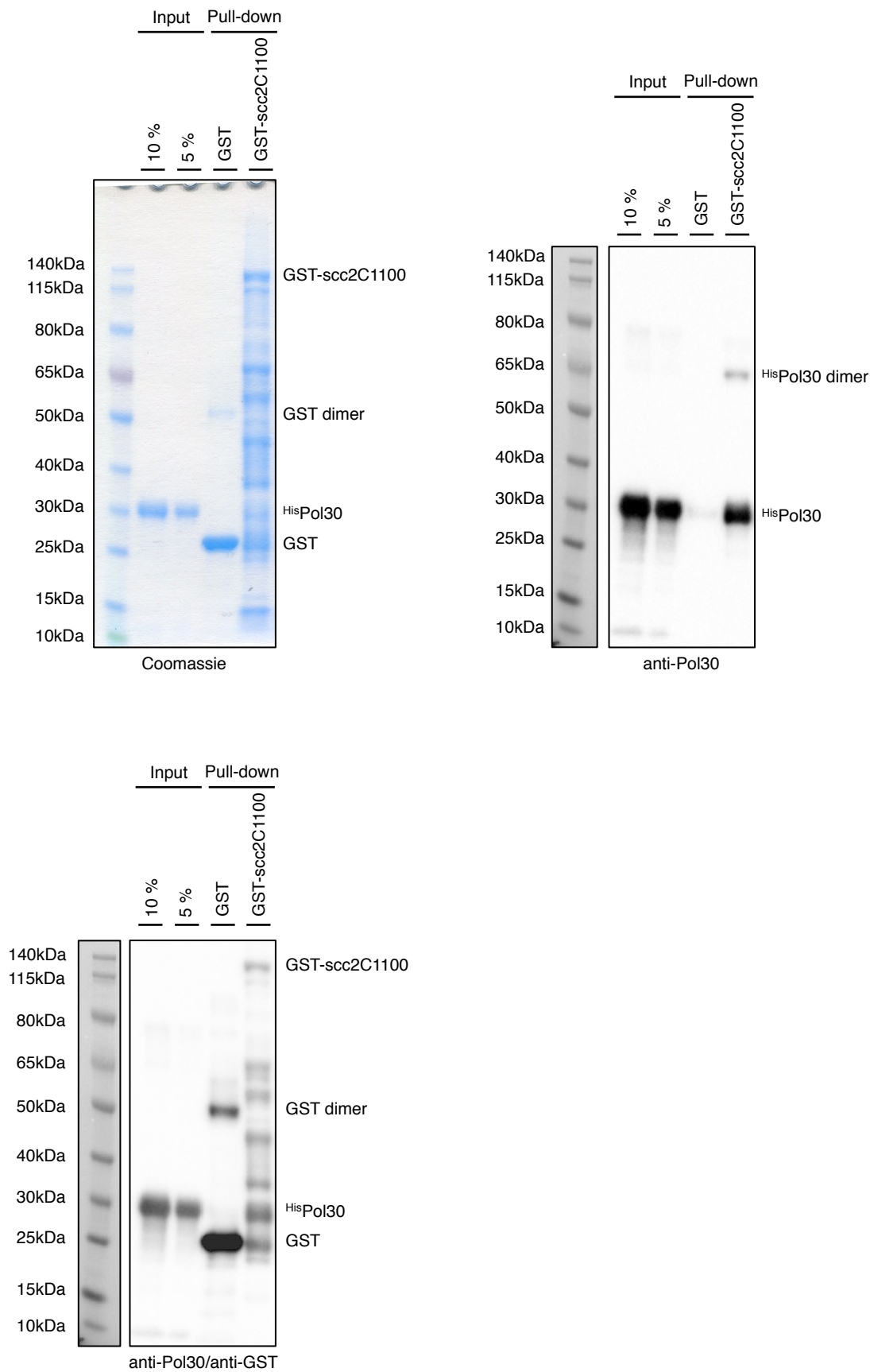

Supplement: Source Data Extended Data Fig. 2 — Unprocessed western blots and gels. [file 41594_2023_1064_MOESM12_ESM.pdf]

Raw Data Extended Data Figure 3

a

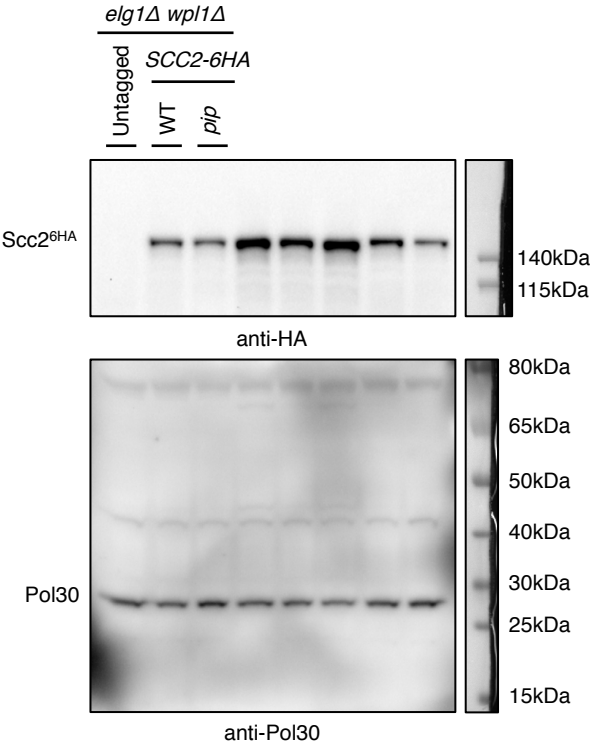

Supplement: Source Data Extended Data Fig. 3 — Unprocessed western blots. [file 41594_2023_1064_MOESM13_ESM.pdf]

Raw Data Extended Data Figure 5

a

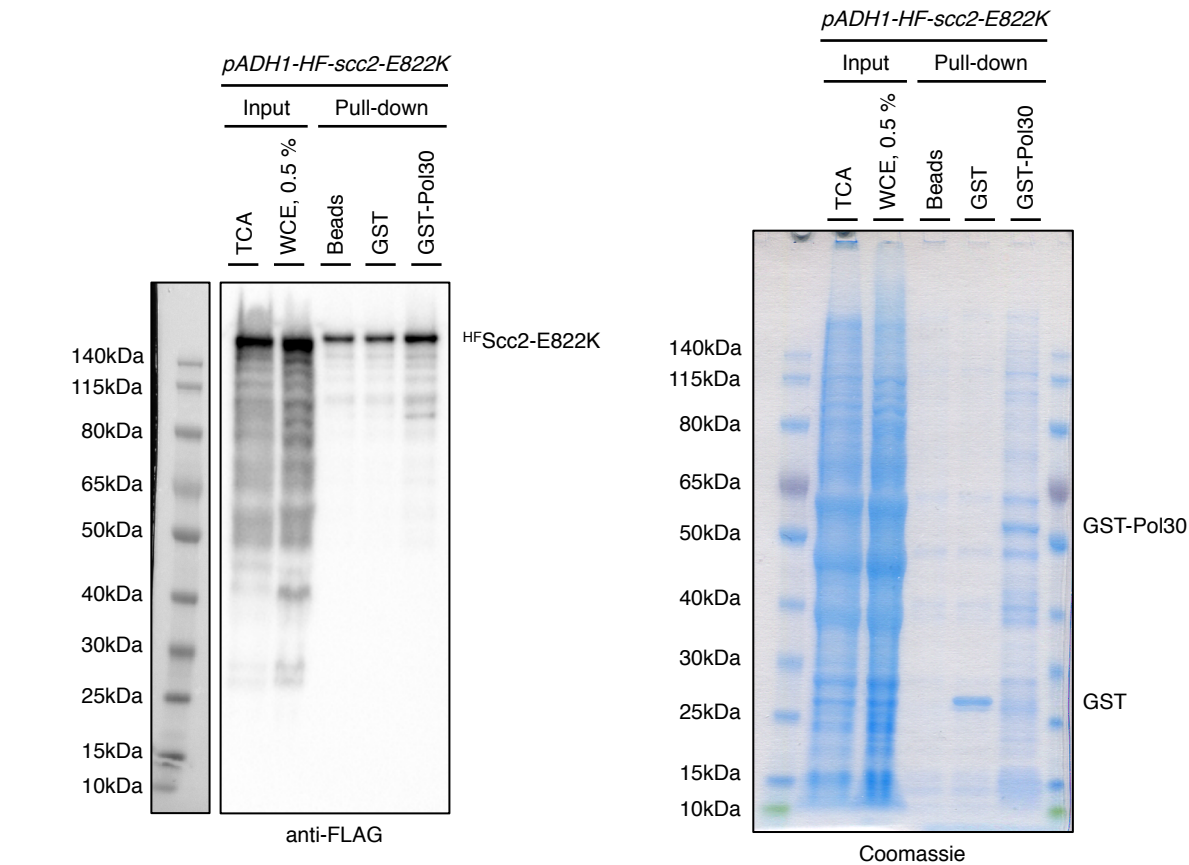

b

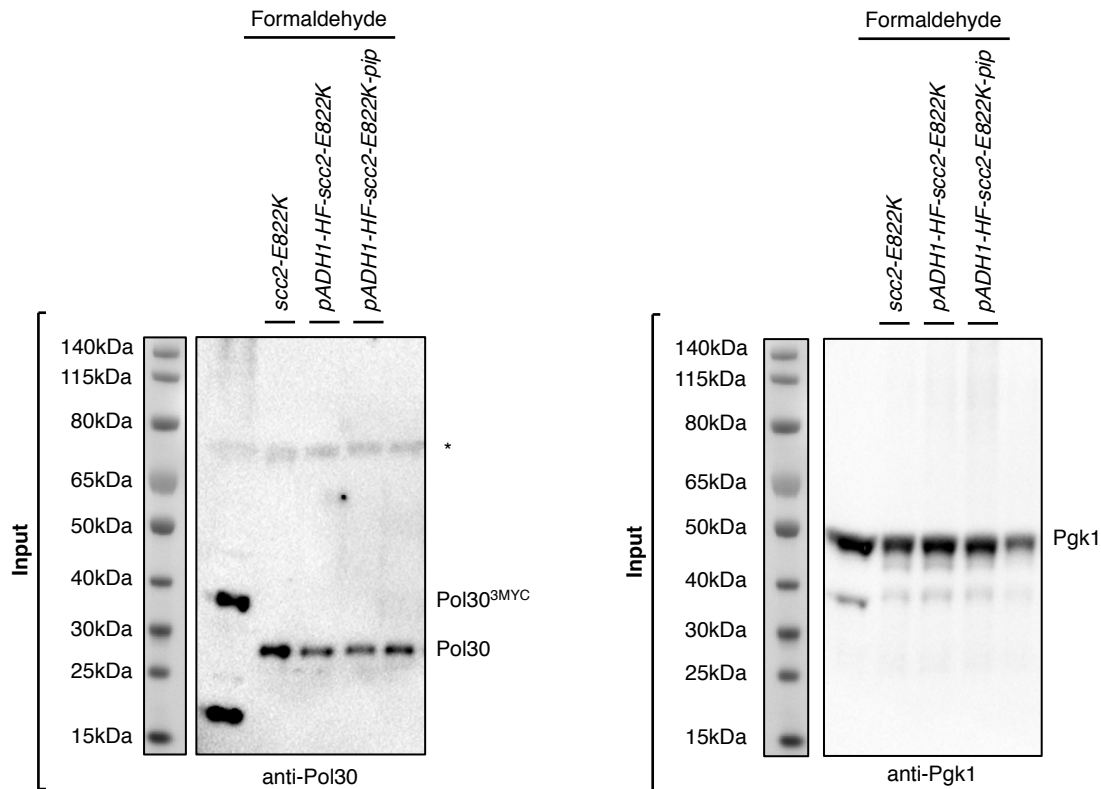

Raw Data Extended Data Figure 5

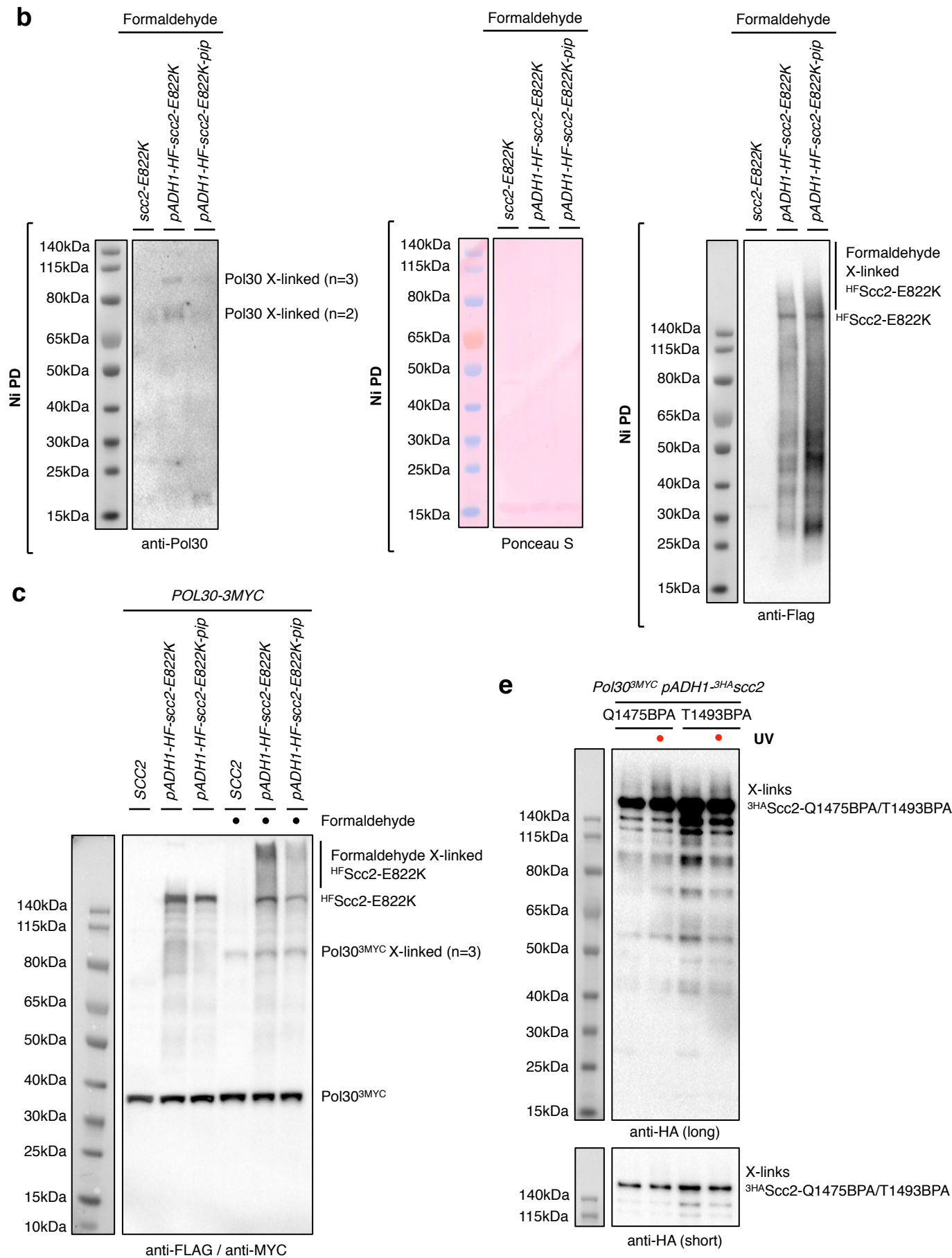

Raw Data Extended Data Figure 5

e

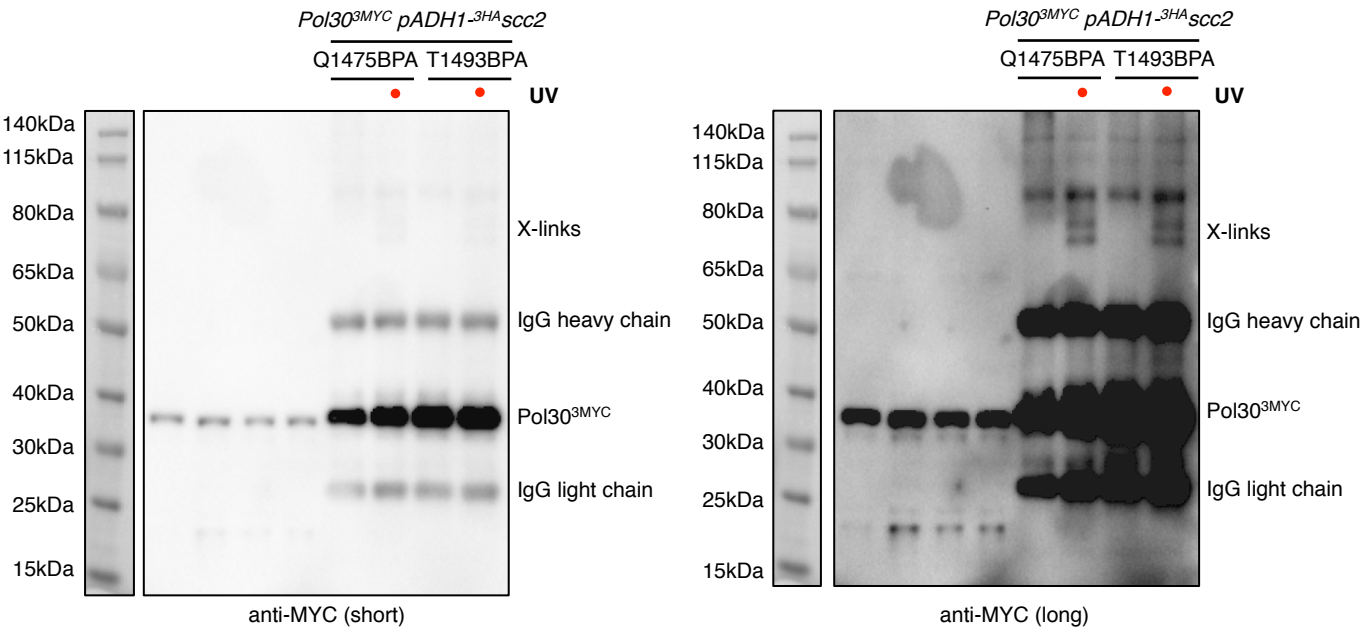

Supplement: Source Data Extended Data Fig. 5 — Unprocessed western blots and gels. [file 41594_2023_1064_MOESM14_ESM.pdf]

Raw Data Extended Data Figure 7

e

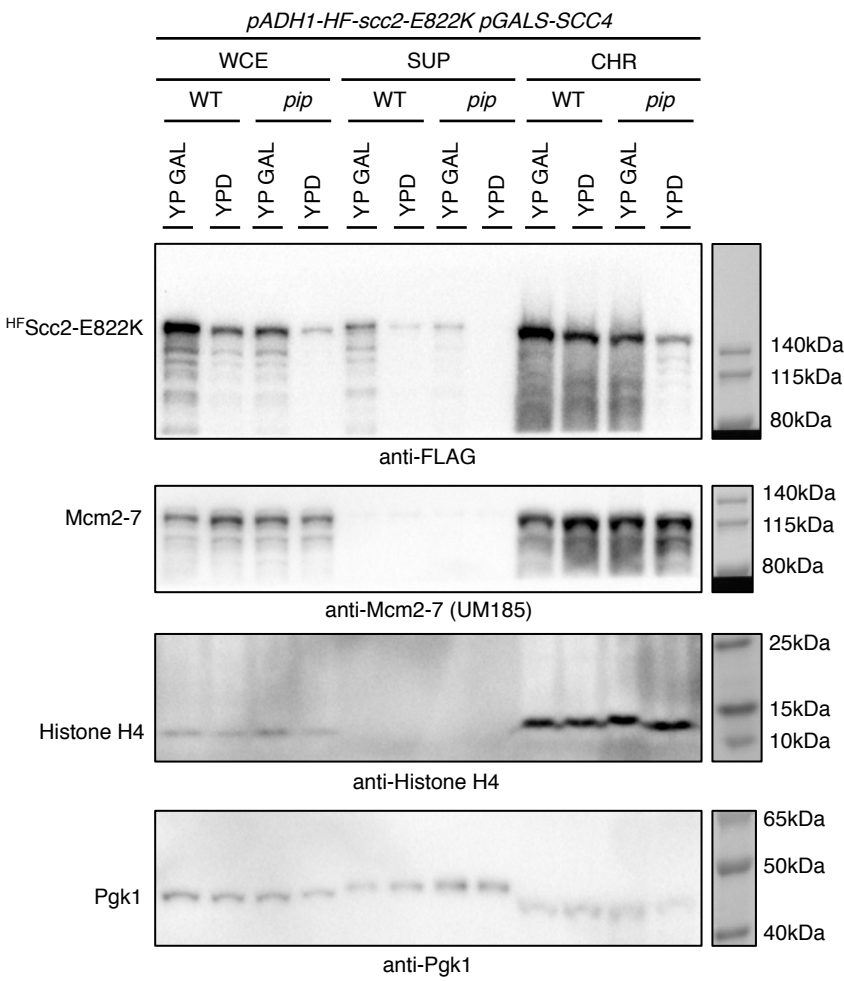

Supplement: Source Data Extended Data Fig. 7 — Unprocessed western blots. [file 41594_2023_1064_MOESM15_ESM.pdf]

**a**

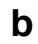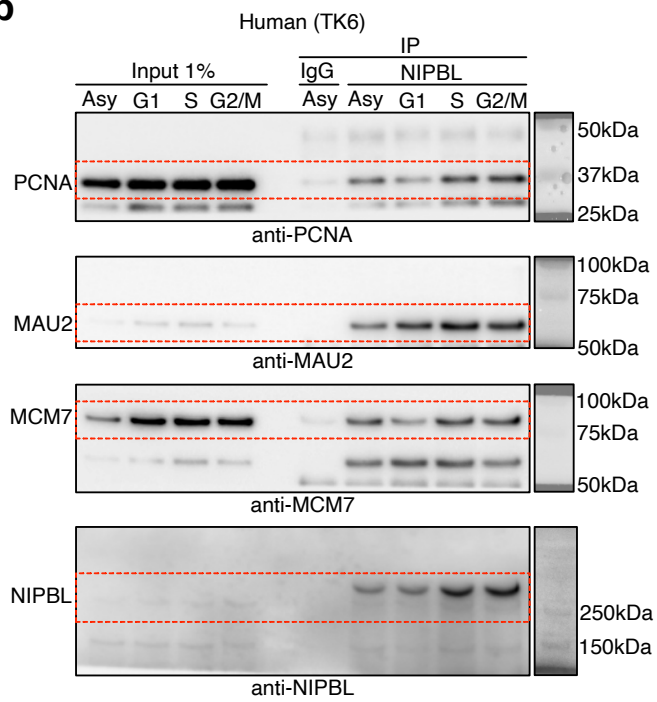

Raw Data Extended Data Figure 8

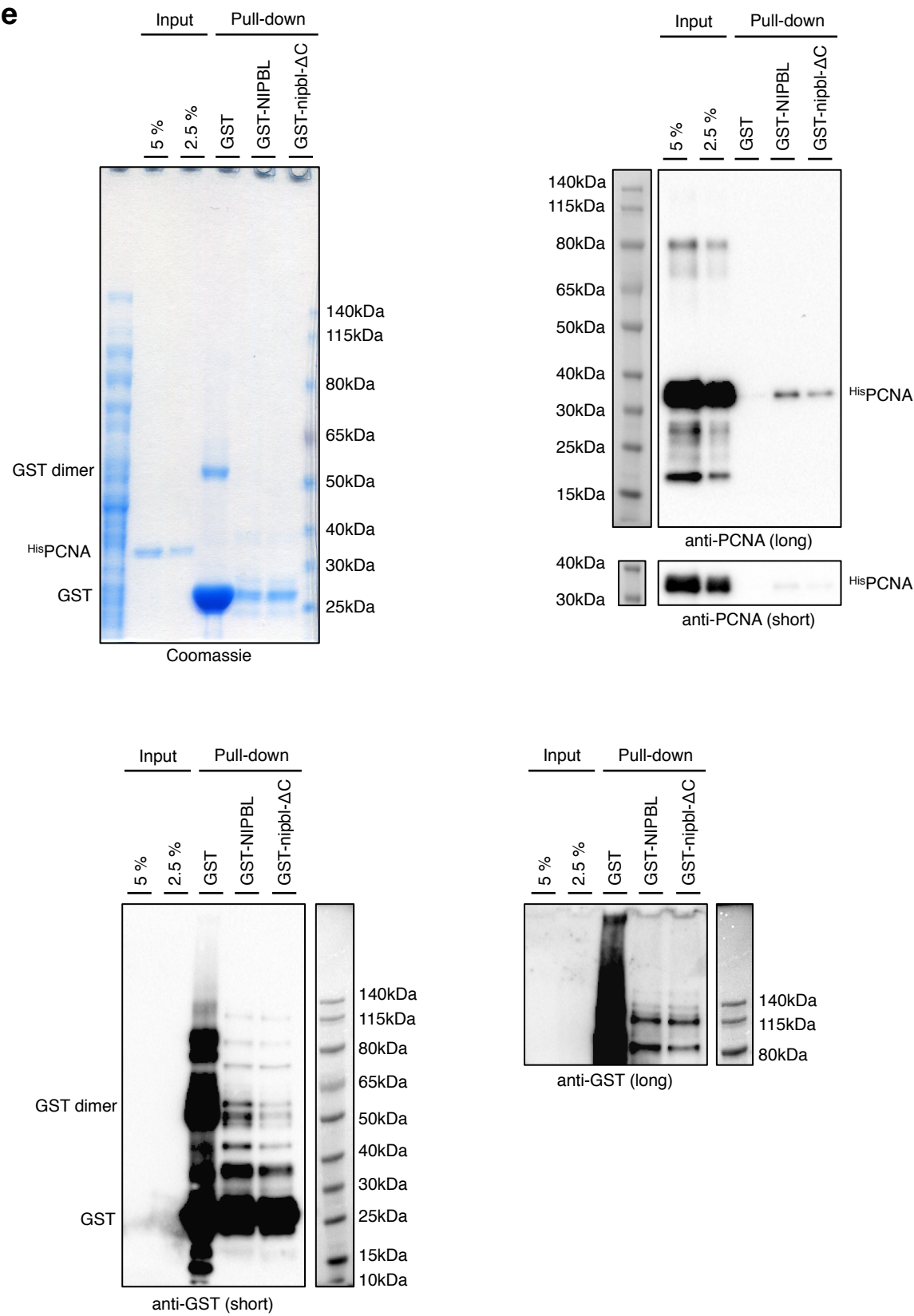

Raw Data Extended Data Figure 8

f

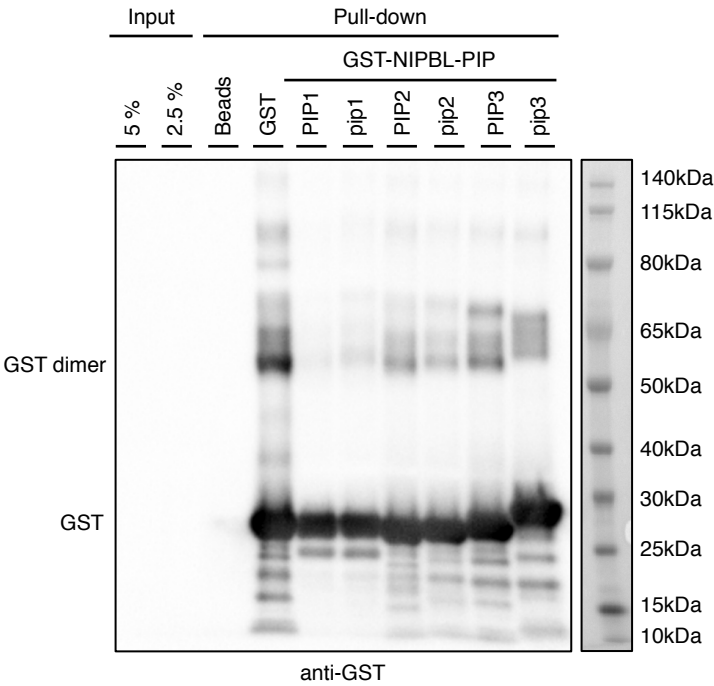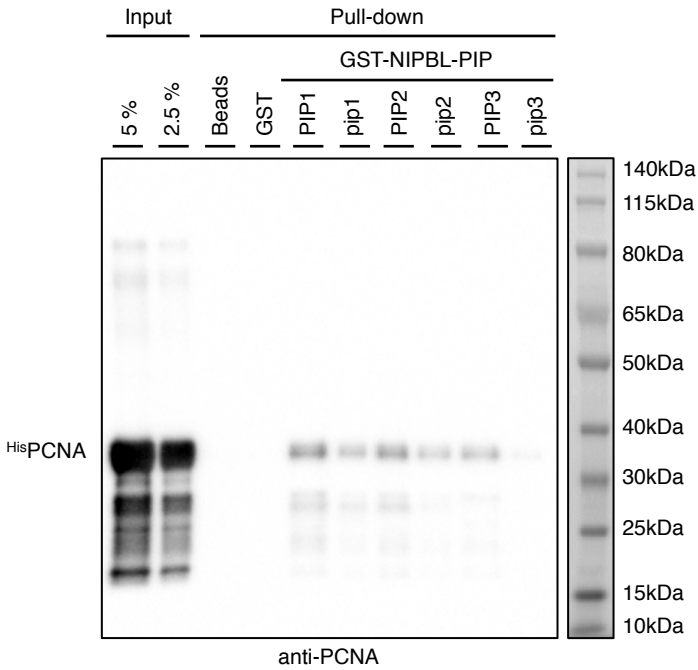

Supplement: Source Data Extended Data Fig. 8 — Unprocessed western blots and gels. [file 41594_2023_1064_MOESM16_ESM.pdf]

Raw Data Extended Data Figure 10

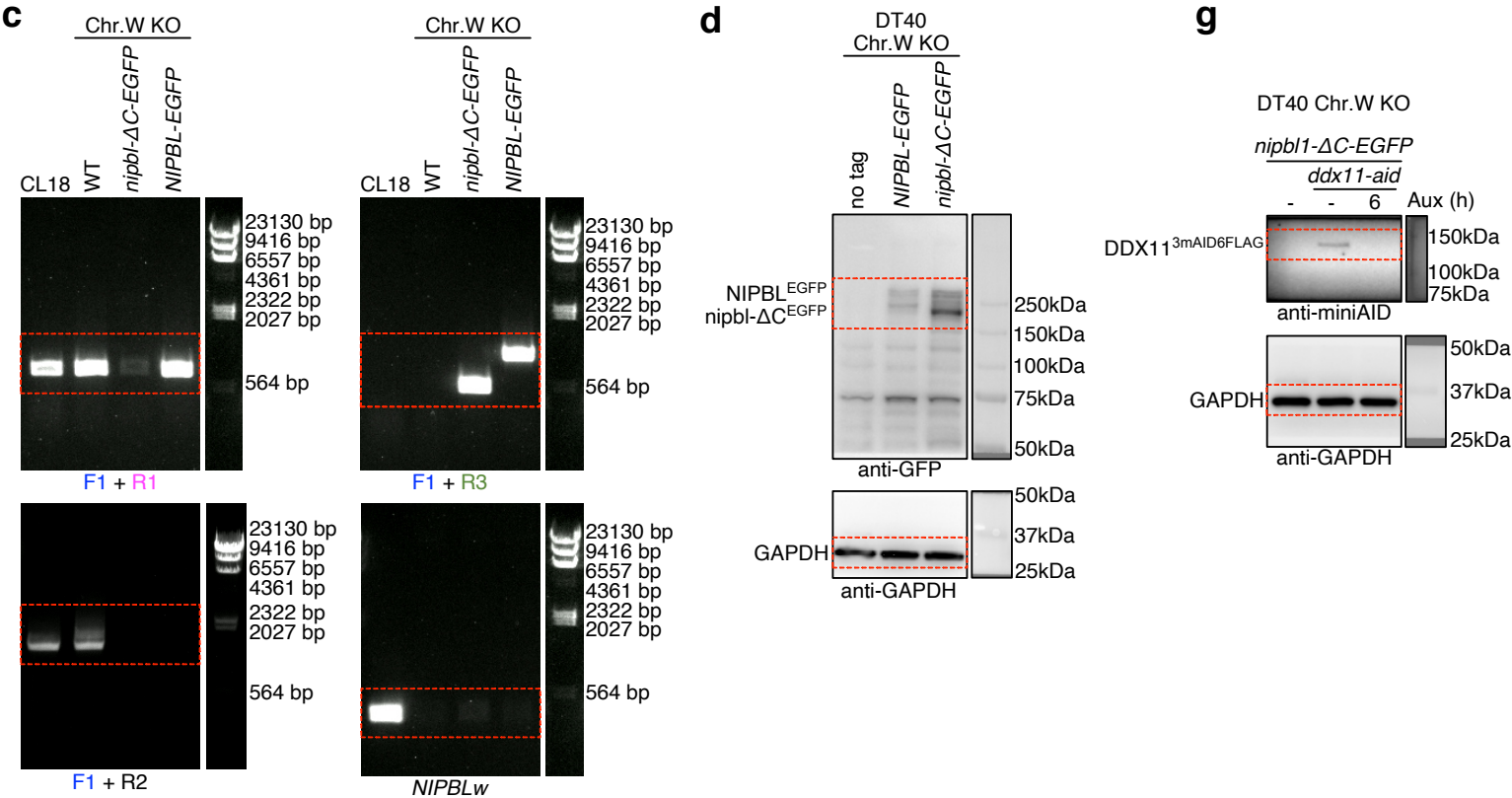

Supplement: Source Data Extended Data Fig. 10 — Unprocessed western blots and gels. [file 41594_2023_1064_MOESM17_ESM.pdf]
